# The neurological damage caused by enterovirus 71 infection is associated with hsa_circ_0069335/miR-29b/PMP22 pathway

**DOI:** 10.1128/jvi.00844-24

**Published:** 2024-12-05

**Authors:** Guangming Liu, Danping Zhu, Kuan Feng, Hongxia Peng, Sida Yang, Li Huang, Peiqing Li

**Affiliations:** 1 Pediatric Emergency Department, Guangzhou Women and Children’s Medical Center, Guangzhou Medical University 26468 , Guangzhou, China; University of Michigan Medical School, Ann Arbor, Michigan, USA

**Keywords:** neurological damage, enterovirus 71, Schwann cells, myelin sheath, PMP22

## Abstract

Enterovirus 71 (EV71) infection is usually accompanied by neurological damage, which is the leading cause of death in children with hand-foot-mouth disease. In this study, we demonstrated that EV71 infection can cause pathological damage in the nervous system, such as neuronal vacuolar degeneration, shrinkage of some neurons, edema of brain tissues in the hippocampus, and a decreased number of Nissl bodies in the infarction area. Also, EV71 infection caused apparent structural damage to Schwann cells, including a decreased number of cytoplasmic organelles and severe damage of rough endoplasmic reticulum and mitochondria. However, the pathological damage was alleviated with the decrease of EV71 viral load. The cell experiment *in vitro* showed that EV71 infection significantly reduced ATP levels and promoted Schwann cell apoptosis, thus inhibiting cell growth. The extended infection time and the decreased viral load resulted in the gradual improvement of cell growth status. Meanwhile, EV71 inhibited the expression of miR-29b and promoted the expression of PMP22 in a time-dependent manner at both mRNA and protein levels, with the most significant change at 36 h of infection. Subsequently, the expression of miR-29b and PMP22 was gradually restored with the reduction of EV71 viral load. In addition, EV71 regulated the expression of hsa_circ_0069335, which could bind and co-localize with miR-29b. Therefore, EV71 infection can cause significant damage to the nervous system and may be related to hsa_circ_0069335/miR-29b/PMP22 pathway. The present study provides a new therapeutic target for neurological damage induced by EV71 infection.

**IMPORTANCE:**

EV71 can cause severe neurological damage and even death, but the mechanism remains unclear. In this study, we exhibited the pathological changes of nervous system in EV71 infection and revealed that the damage degree was consistent with the EV71 viral load. From the molecular perspective, EV71 infection up-regulated the PMP22 expression in Schwann cells, which is accompanied by apparent structural damage of Schwann cells and myelin sheaths. Furthermore, EV71 promoted the expression of PMP22 and inhibited the expression of miR-29b in a time-dependent manner, with the most significant change at 36 h of infection. Otherwise, the hsa_circ_0069335, which binds and co-localizes with miR-29b, was also regulated by EV71 infection. The hsa_circ_0069335/miR-29b/PMP22 axis may be a potential molecular mechanism involved in EV71 infection-induced fatal neuronal damage. Drug development targeting this pathway may bring clinical improvement of EV71-infected patients.

## INTRODUCTION

Enterovirus 71 (EV71) is a single-stranded RNA virus and the main pathogenic factor of hand-foot-mouth disease (HFMD), which has caused several large outbreaks worldwide and created a huge disease burden in the world, especially in the Asia-Pacific region (1–3). Although the main symptoms of HFMD are ulcerative blisters in the mouth and viral rashes in the hands and feet (4–6), a small number of patients may develop cardiopulmonary or nervous system complications, including aseptic meningitis, encephalitis, acute transverse myelitis, polio-like syndrome, Guillain-barre syndrome, acute cerebellar ataxia, and benign intracranial hypertension, which may be fatal (7, 8). Considering that most HFMD patients are asymptomatic, early diagnosis and treatment of nerve injury may be challenging, so it is necessary to search for reliable biomarkers to assist in making early clinical decisions.

Some neurological complications mentioned above are closely related to the demyelinating process (9, 10). The myelin sheath is a multilayered structure that wraps around axons and effectively transmits nerve impulses. Myelin abnormalities affect the transmission of nerve impulses and lead to axonal degeneration, which is the pathological basis of various neurological diseases (11). The myelin sheath of the peripheral nervous system is mainly composed of Schwann cells, which surround nerve axons to form myelinated nerve fibers and provide protection and nutrition for axons to maintain normal physiological functions. Impaired Schwann cell function eventually leads to demyelinating lesions and axon loss (12, 13).

Peripheral myelin protein 22 (PMP22), a transmembrane glycoprotein component of myelin, is mainly expressed in Schwann cells of peripheral nerves, accounting for 2%–5% of the total myelin protein content and having a vital role in the development of nerve myelination and the repair of demyelinating axons through myelin regeneration in diseased nerves (14). Our previous studies have shown that overexpression of EV71 structural virus protein 1 (VP1) can enhance endoplasmic reticulum stress, thereby promoting PMP22 expression and increasing mouse Schwann cell autophagy (15). Therefore, VP1 may affect the function of Schwann cells by regulating the expression of PMP22 and ultimately affecting myelin formation. However, its mechanism remains unclear.

Some studies have found that miR-29a negatively regulates the expression of PMP22 (16, 17). In recent years, it has been shown that non-coding circular RNA (circRNA) has the adsorption effect of micro-RNA (miRNA) sponge (18), which can block or reduce the inhibitory effect of disease-related miRNA on genes, thus promoting the expression of target genes and creating the conditions and advantages to become a new diagnostic marker for diseases (19). At present, specific expression of circRNAs has been found in various neurological diseases (20), and studies have also shown that circRNAs participate in Schwann cells (21). However, until now, the relationship between EV71 and circRNAs, and whether circRNAs affect myelination, has not been studied. Therefore, this study searched for circRNAs associated with miR-29/PMP22 through sequencing, aiming to find accurate qualitative and quantitative diagnostic and evaluation methods for nerve myelin sheath damage during EV71 infection and assist clinical timely and targeted treatment decisions for neuroprotection.

## MATERIALS AND METHODS

### Cell culture and virus

African green monkey kidney epithelial cell line, Vero cells (Cat no. CCL-81; ATCC), was cultured in high-glucose Dulbecco’s modified Eagle’s medium (DMEM) (Invitrogen, Carlsbad, CA, USA) supplemented with 10% fetal bovine serum (FBS; Hyclone, Logan, UT, USA) and at 37°C with 5% CO_2_. Enterovirus A71 strain GZ203KL21/GD/CHN/2010 is preserved in our laboratory and was isolated from an HFMD patient in Guangzhou, China (GenBank accession no. MF362981). EV-A71 was amplified in Vero cells after infection. Virus was harvested at 3 days post-infection, and the viral titers were routinely determined in Vero cells by the microplate dilution method and calculated by the Karber method.

### Infection of mice with EV71

Sixteen pregnant ICR mice were purchased from the Laboratory Animal Center of Guangzhou University of Traditional Chinese Medicine and kept in separate ventilated and specific pathogen-free cages. Twelve mice were randomly selected for EV71 infection, and 4 of them were selected every 24 h for subsequent experimental testing. The ICR neonatal mice were intracranially inoculated with Enterovirus A71 strain, and the experimental procedure was as follows: after anesthesia, the holes were drilled on the anterior fontanelle in mice, which were then stereotaxically injected 2 µL of virus (2 × 10^9^ /mL) suspension into the brain at a rate of 0.2 µL/min.

### Hematoxylin-eosin staining

The brain tissues of euthanized mice were fixed with 4% paraformaldehyde for more than 24 h, followed by dehydration, embedding, and sectioning. After the slices were dewaxed, nuclear staining was performed with hematoxylin, followed by cytoplasmic staining with eosin. After dehydration, the slices were sealed with neutral gum, and the histopathological changes were analyzed by photographing.

### Nissl staining

After dewaxing, paraffin sections were stained with toluidine blue for 5 min and then washed, and 1% glacial acetic acid was added for differentiation. Subsequently, the slices were treated with xylene for 5 min, then sealed with neutral gum. Finally, microscopic examination and image acquisition analysis were performed.

### Transmission electron microscopy detection

Tissue samples were sequentially fixed at 4°C for 2 h with 2.5% glutaraldehyde and 1% osmic acid prepared with sodium cacodylate buffer, followed by sequentially dehydrated with graded concentrations of ethanol and acetone. After soaking with 1:1 acetone and resin, the sample was embedded and polymerized with an embedding agent. Ultrathin sections were then made and stained with uranium acetate solution at room temperature for 10–20 min. After washing, sections were stained with lead citrate solution for 15 min and finally observed with a transmission electron microscope (Hitachi H-7500, Japan).

### Immunofluorescence

Paraffin sections were dewaxed with xylene and ethanol, after which the citric acid antigen repair buffer was added for antigen repair. Next, the sections were treated with autofluorescence quencher for 5 min and incubated with bovine serum albumin (BSA) for 30 min. After removing the blocking solution, the sections were incubated overnight at 4°C with anti-enterovirus 71 antibody (1:500; Abcam, China), and then FITC-labeled secondary antibody (1:200; Bioss, China) for 50 min at room temperature in the dark. Finally, the cell nucleus was restained with 4,6-diamidino-2-phenylindole (DAPI), and the sections were sealed with an anti-fluorescence quenching sealing agent and observed under a fluorescence microscope. Similar methods were used to detect PMP22 and EV71 levels in mouse Schwann cells, and the cell slides were incubated with anti-PMP22 (1:500; Bioss, China) or anti-enterovirus 71 antibody at 4°C overnight.

### Primary isolation and culture of Schwann cells

**Fig 1:**
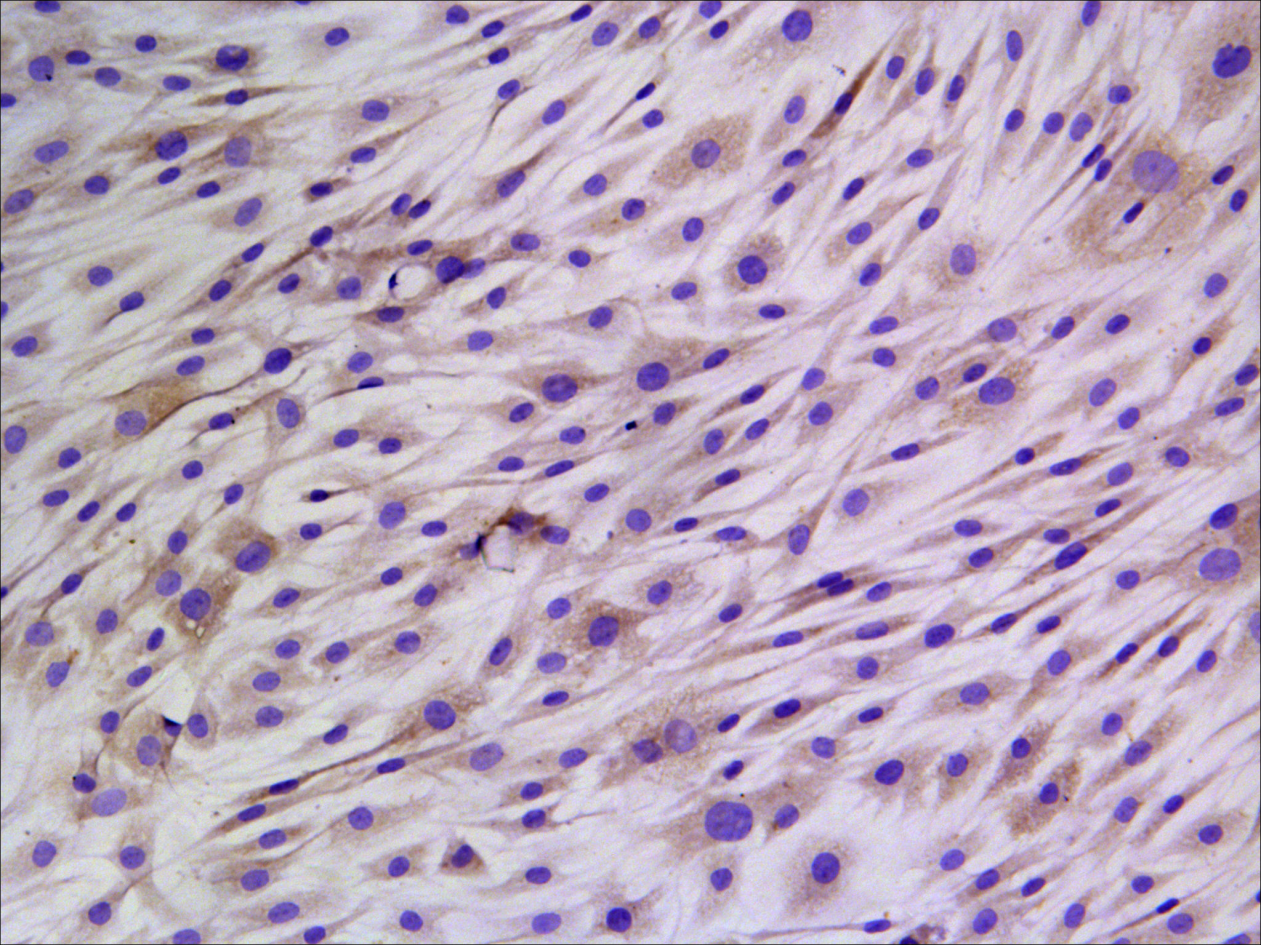
Immunohistochemical staining of S-100 in cultured Schwann cells (100). The cell staining was performed with DAB, followed by nuclear staining with hematoxylin (blue). The S-100-positive cells are brownish yellow.

Ganglia were isolated from the brains of ICR mice 1–3 days after birth, digested with trypsin, and then cultured with DMEM (Invitrogen, Carlsbad, CA, USA) containing 10% FBS (Hyclone, Logan, UT, USA) and 2 mM glutamine for 30 min. After removing the adherent fibroblasts, 2 mL cell suspension with a planting density of 0.2 × 10^5^ cells/mL was seeded into a 35 mm plastic culture dish coated with rat-tail collagen and cultured at 37°C with 10% CO_2_. On the third day of cell culture, the cell division inhibitors 5-fluoro-2-deoxyuridine (15 µg/mL) and uridine (35 µg/mL) were added to further remove the fibroblasts, respectively. After 48 h of continuous culture, the fresh culture medium was replaced, and then changed twice a week, each time replacing half of the fresh culture medium. Schwann cells with a high purity level of 95% were obtained after 15 days of culture and were positive by S-100 immunohistochemical staining (Table 1; Fig. 1).

**TABLE 1:** Proportion of S-100 positive cells in cultured mouse Schwann cells

Mouse Schwann cell	S-100-positive cells	Total cells	Proportion of S-100-positive cells (%)	Mean ± SD
Cell 1	103	111	92.7	0.95 ± 0.01
Cell 2	108	113	95.6	
Cell 3	96	100	96.0	

### MTS assay

A total of 100 µL cells with a density of 1 × 105 cells/mL were seeded into a 96-well plate. After cell attachment, the cells at various time points (0, 24, 48, and 72 h) were collected to detect cell proliferation using CellTiter 96 AQueous One Solution Cell Proliferation Assay (Promega, Beijing, China). After incubating with the detection solution for 4 h, the optical density value at 450 nm was read with a microplate reader (multiscan MK3, Thermo Fisher Scientific)

### Measurement of intracellular ATP content

The intracellular ATP content was detected using an enhanced ATP assay kit (Beyotime, Shanghai, China). First, 200 µL lysis solution was added to each well of the six-well plate for cell lysis, after which the supernatant was collected by centrifugation at 4°C and 12,000 g for 5 min. The supernatant was added with 100 µL of ATP detection working solution and placed at room temperature for 3–5 min, and then the relative light unit value was measured using a luminometer.

### Detection of early and late cell apoptosis

Cell apoptosis was detected using the Annexin V-FITC apoptosis assay kit (KeyGEN, Nanjing, China) and the DeadEnd Fluorometric TUNEL System (Promega), respectively. First, cells (including shed cells in the medium) were collected and resuspended, and the cell density was adjusted to 1 × 10^6^ cells/mL. Then, cell suspension containing 5 × 10^5^ cells was mixed with 1.25 µL Annexin V-FITC and placed at room temperature away from light for 15 min. Next, the cell precipitate obtained by centrifugation at 1,000 × g for 5 min was resuspended, mixed with 10 µL propidium iodide, and then analyzed by flow cytometry. The experimental procedure of TdT-mediated dUTP Nick-End Labeling (TUNEL) to detect apoptosis was as follows: cells were fixed with 4% formaldehyde solution at 4°C for 25 min and then incubated with 0.2% Triton X-100 at room temperature for 5 min; after washing with phosphate buffered saline (PBS), the cells were equilibrated with 100 µL Equilibration Buffer at room temperature for 10 min and then incubated with 50 µL TdT working solution at 37°C for 60 min in the dark. Next, the cells were washed with 2× saline sodium citrate buffer (SSC) solution and PBS, respectively, and incubated with DAPI staining solution at room temperature for 10 min in the dark; after sealing with fluorescent anti-quenching agent, the cells were observed under a fluorescence microscope.

### Fluorescence quantitative PCR

Total RNA was extracted from mouse Schwann cells using Trizol (Invitrogen, Carlsbad, CA, USA), after which the RNA was reverse-transcribed into cDNA using EasyScript First-Strand cDNA Synthesis SuperMix (TransGen, Beijing, China), while specific primers were added for reverse transcription to detect miR-29b, PMP22, and cicrRNA. Quantitative PCR was performed using SYBR Green qPCR SuperMix (Vazyme, Nanjing, China) with three replicates for each sample. The relative expression level of each gene was calculated using the 2−ΔΔCt method.

### Western blot

Mouse Schwann cells were lysed with radioimmunoprecipitation assay (RIPA) buffer (Beyotime) to obtain total protein. Then, the protein concentration within the lysates was assessed using the Micro BCA Protein Assay Kit (Thermo Fisher Scientific, Waltham, MA, USA). Subsequently, the proteins were subjected to SDS-PAGE and membrane transfer. After washing off the transfer solution, the membrane was incubated with anti-PMP22 antibody (1:500; Bioss) or anti-glyceraldehyde-3-phosphate dehydrogenase (GAPDH) antibody (1:1,000; Bioss) overnight at 4°C, followed by the addition of horseradish peroxidase-labeled secondary antibody (1:4,000; SouthernBiotech, Birmingham, AL, USA) at room temperature for 2 h. Next, protein bands were detected using BeyoECL Plus (Beyotime) and analyzed by a gel image processing system. Densitometry analysis was performed using Quantity One (Bio-Rad, Hercules, CA, USA), and the band intensities were normalized to those of GAPDH.

### Luciferase reporter assay

Hsa_circ_0069335 sequence was cloned into psiCHECK-2 vector (Promega), and the potential miR-29b-binding site was mutated so that the expression vector containing wild type or mutant hsa_circ_0069335 sequence was obtained. These vectors were co-transfected with related miRNAs, and then the Luciferase Reporter Assay System (Promega) was used to detect luciferase activity.

### Fluorescence *in situ* hybridization

The cell slides were incubated with PBS containing 0.3% Trition X-100 at room temperature for 20 min, followed by 4% paraformaldehyde at room temperature for 20 min. After washing with PBS, the slides were incubated with RNA hybridization buffer at 55°C for 2 h, and then 20 µL denatured probe working solution was added and hybridized at 37°C overnight. The slides were washed with PBS three times and stained with DAPI at room temperature for 5 min. Finally, the slides were sealed with the fluorescent anti-quenching agent and examined under the microscope.

### Statistical analysis

All experimental data were expressed as mean and SD. Statistical analysis was performed using SPSS 22.0 software. Comparisons between two groups were performed using the least significance difference (LSD)-t test, and comparisons between multiple groups were performed using one-way analysis of variance. *P* < 0.05 represented a statistically significant difference.

## RESULTS

### EV71 infection causes significant pathological changes in the nervous system

**Fig 2:**
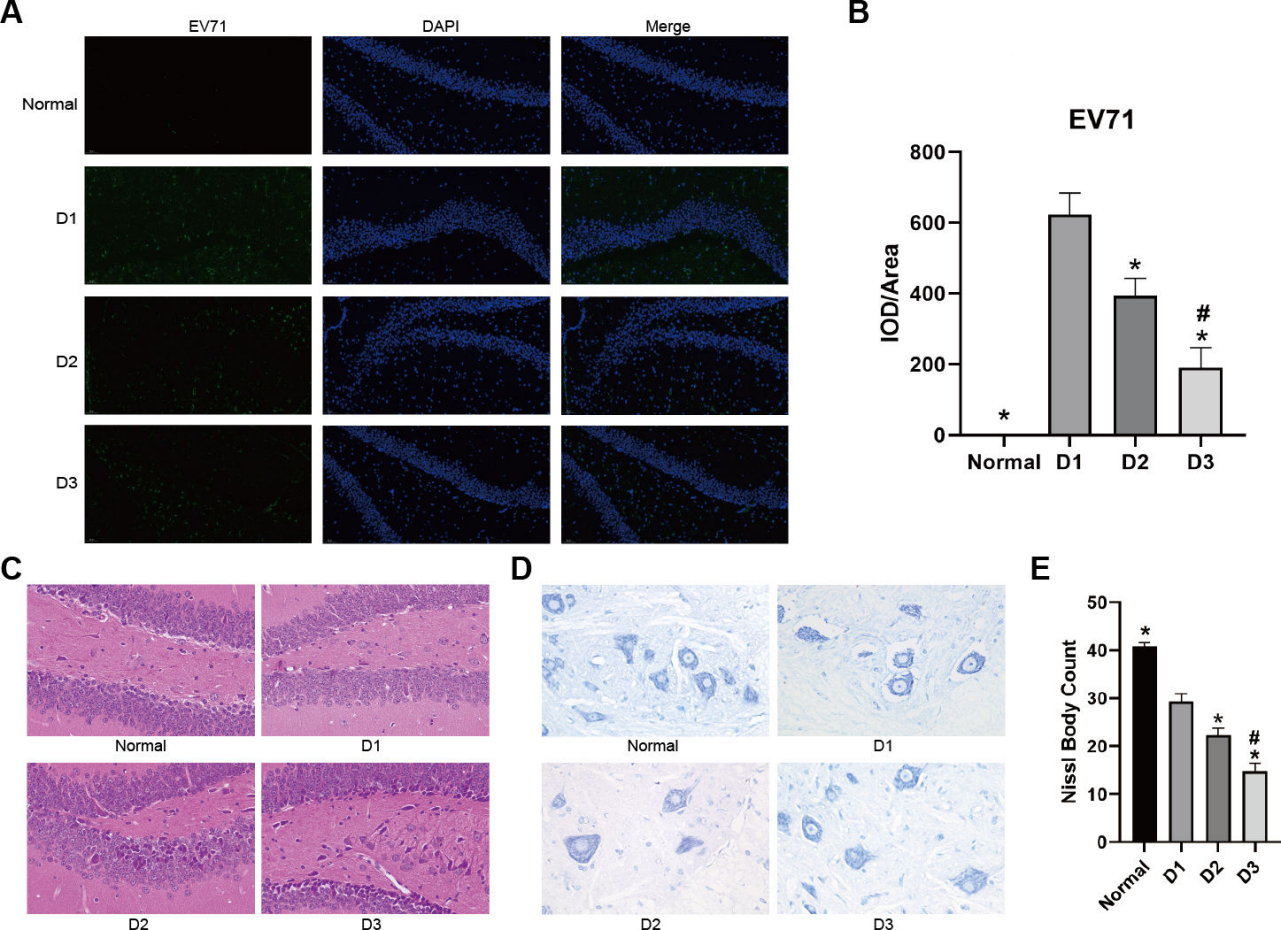
Pathological changes of brain tissues after EV71 infection in mice. The ICR neonatal mice were intracranially inoculated with Enterovirus A71 strain, and the brain tissues were analyzed at 1–3 days post-infection. (**A**) Representative images of EV71 stain by immunofluorescence assay. Laser scanning confocal microscopy of brain tissue stained with nuclear stain (DAPI, fluorescing blue), EV71 stain (fluorescing green). Magnification, 20×. (**B**) Quantification of relative EV71 expression level by immunofluorescence staining analysis. *P < 0.05 vs D1. # *P* < 0.05 D2 vs D3. (**C**) Hematoxylin-eosin staining to detect the pathological changes of brain tissue. Magnification, 400×. (**D**) Nissl staining to detect Nissl bodies of brain tissue. Magnification, 400×. (**E**) Quantification analysis of Nissl bodies in brain tissue. *P < 0.05 vs D1. # *P* < 0.05 D2 vs D3.

In order to observe the damage caused by EV71 in mice, we injected EV71 into mice and observed the changes in the nervous system. Immunofluorescence detection of EV71-infected mice showed that the fluorescence intensity in the hippocampus was most significant on 1 day of EV71 infection and then decreased with the increase of infection time (Fig. 2A and B), which indicated decreased viral load with prolonged infection time. Hematoxylin-eosin detection of the hippocampus showed that EV71 infection could lead to neuronal vacuolar degeneration, shrinkage of some neurons, and edema of brain tissues (Fig. 2C). Simultaneously, Nissl body detection revealed that EV71 infection decreased the number of Nissl bodies in the infarction area (Fig. 2D and E).

**Fig 3:**
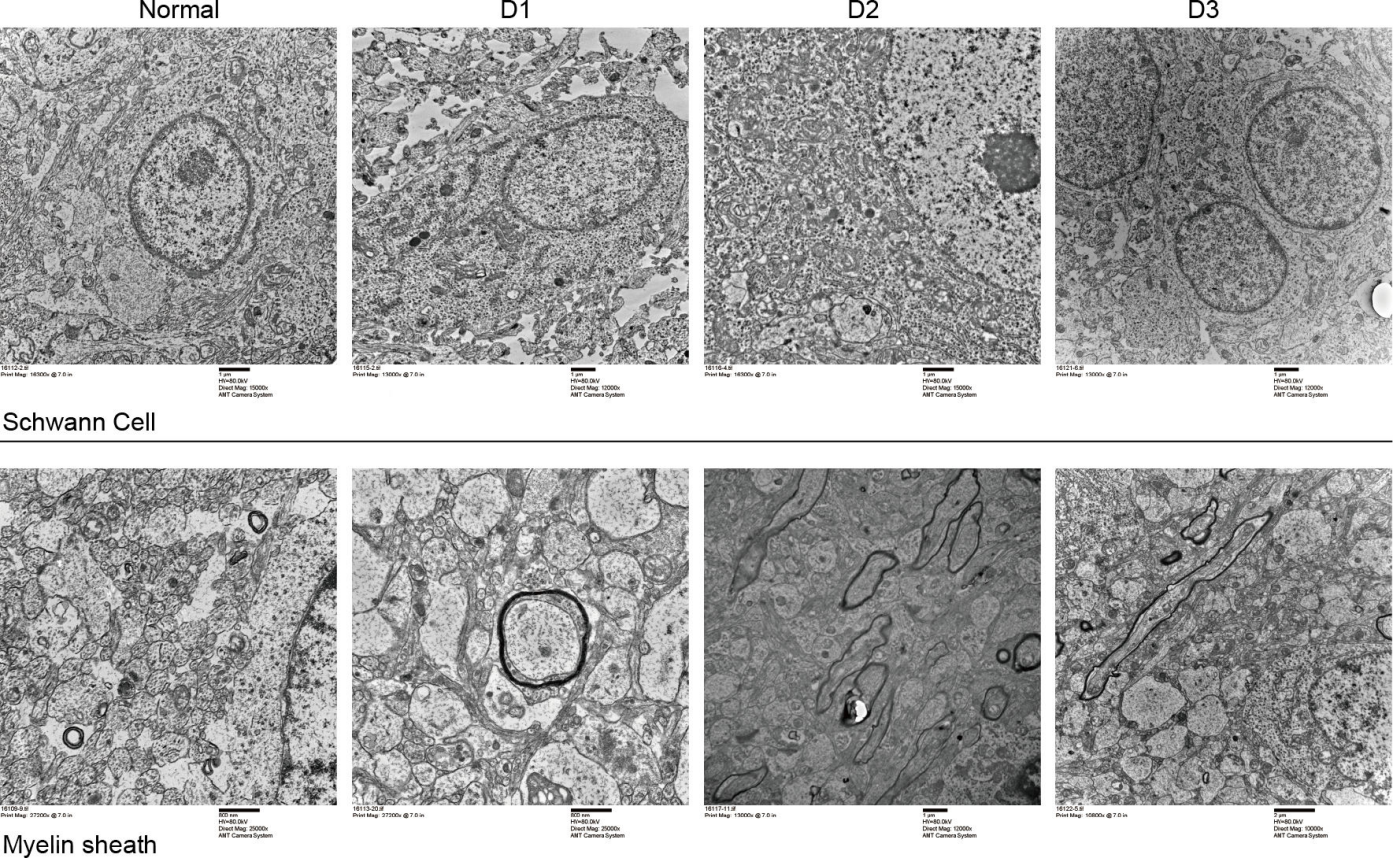
Pathological changes of Schwann cells and myelin sheaths in EV71-infected mice detected by electron microscopy. The ICR neonatal mice were intracranially inoculated with Enterovirus A71 strain, and the pathological changes of Schwann cells and myelin sheaths were observed by electron microscopy examination at 1–3 days post-infection. EV71-infected induced swollen Schwann cells and apparent damages of cytoplasmic organelles, such as expanded rough endoplasmic reticulum with degranulation, severely swollen mitochondria and broken and dissolved mitochondrial cristae. Also EV71 infection decreased the number of intercellular myelin sheaths, dimmed the structure of myelin lamella, and lost the morphology of myelinated nerve fibers.

Next, structural changes of Schwann cells and myelin sheaths were detected by electron microscopy. EV71 infection resulted in apparent structural damage of cells, and some cells were swollen and dissolved; the number of cytoplasmic organelles was decreased, the rough endoplasmic reticulum was expanded with degranulation, the mitochondria were severely swollen, and the mitochondrial cristae were broken and dissolved (Fig. 3). However, with the decrease of EV71 viral load, the degree of cell structural damage was reduced. EV71 non-infected myelin sheaths had complete morphology, dense structure, and clear boundaries; while in EV71-infected myelin sheaths, the number of intercellular myelin sheaths was decreased, the structure of myelin lamella was not clear, the morphology of myelinated nerve fibers was irregular and loosely arranged, and the thickness of myelin sheaths was significantly thinner (Fig. 3). Nevertheless, the pathological changes of myelin sheaths were improved as the EV71 viral load decreased. These data indicate that with the reduction of EV71 viral load, the pathological damage of brain tissues is gradually reduced.

### EV71 infection inhibits Schwann cell growth

**Fig 4:**
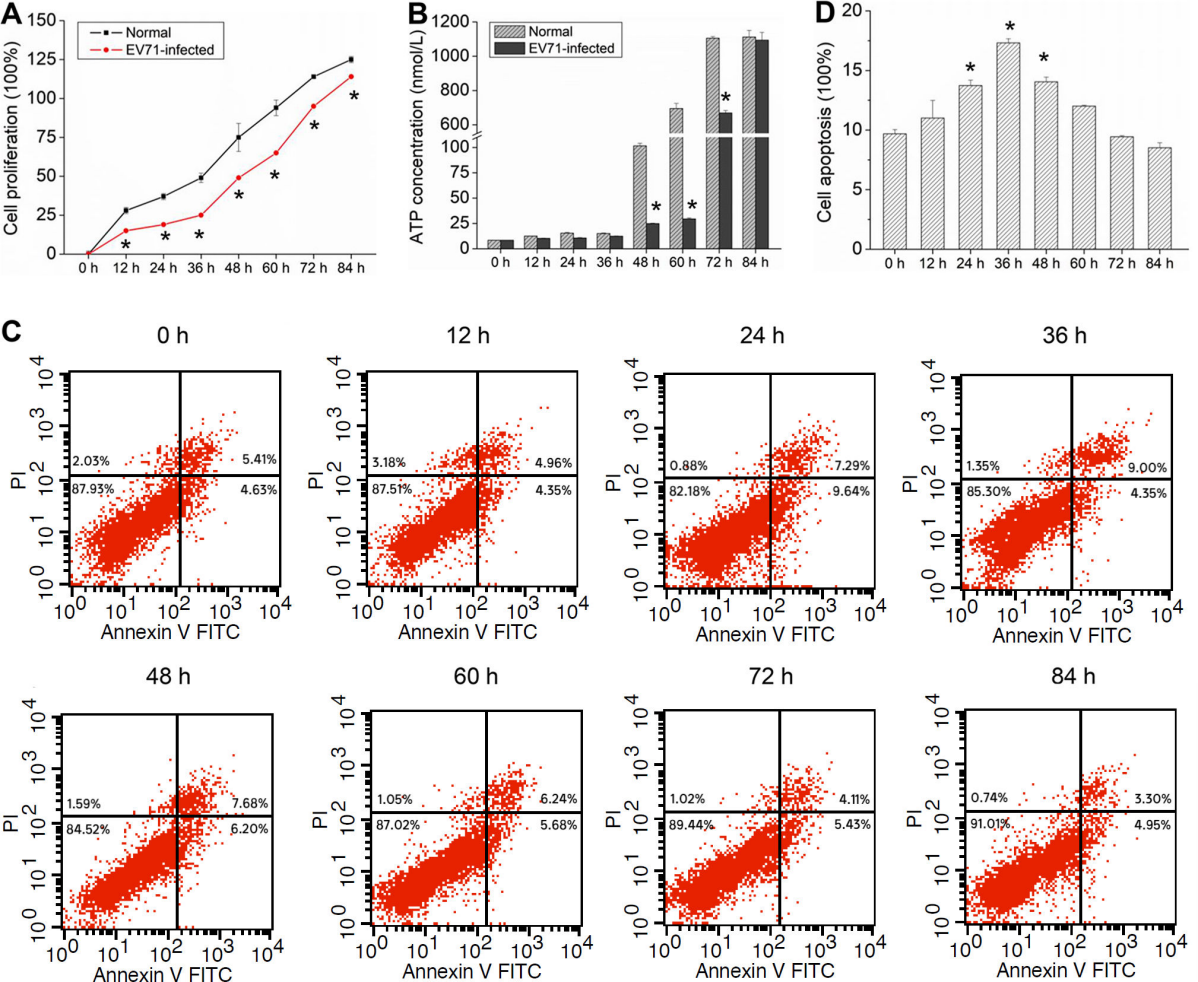
EV71 inhibits the growth of Schwann cells. Schwann cells were collected after EV71 infection at a series of times from 0 to 84 h. (**A**) Growth curve of Schwann cells and the cell proliferation rate were evaluated by the MTS assay. (**B**) Intracellular ATP content of Schwann cells detected by ATP assay kit. (**C**) Cell apoptosis of Schwann cells detected by flow cytometry analysis. The lower left quadrant shows living cells (Annexin V -/PI -). The lower right quadrant shows the early apoptotic cells (Annexin V + /PI-), and the upper right quadrant represents the late apoptotic cells and necrotic cells (Annexin V + /PI+). (**D**) The apoptotic cell proportion of Schwann cells. The proportion was represented by the percentage of Annexin V positive cells. *P < 0.05 vs normal or 0 h.

**Fig 5:**
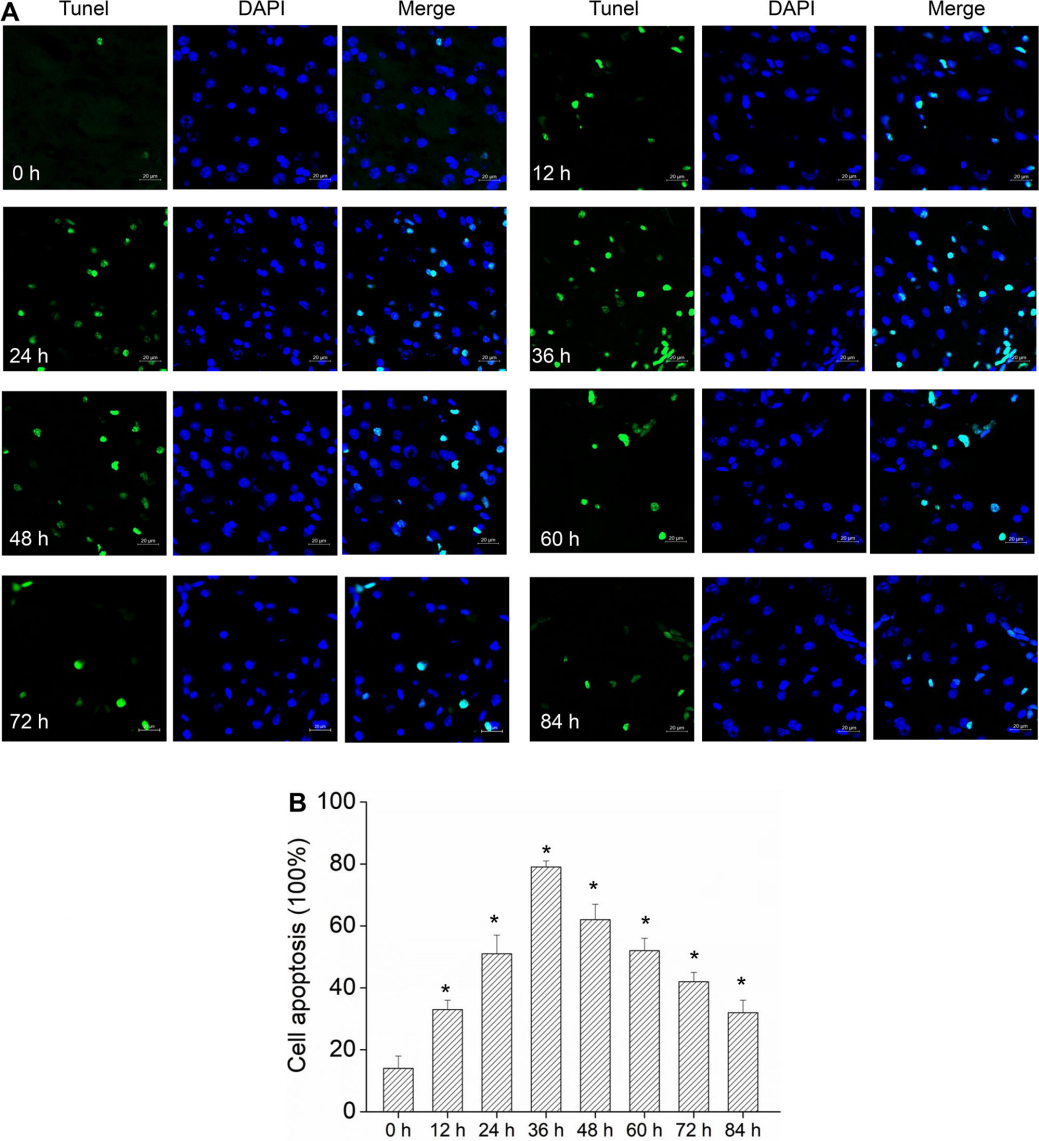
EV71 enhances the apoptosis of Schwann cells. Schwann cells were collected after EV71 infection at a series of times from 0 to 84 h. (**A**) Fluorescence microscope observation of Schwann cells. DNA fragmentation was assessed by TUNEL assay; green-staining cells have DNA fragmentation. Nuclear (DAPI) was stained by blue fluorescence. Scale bars are 20 µm. (**B**) The apoptotic cell proportion of Schwann cells. The proportion was represented by the percentage of DNA fragmentation. *P < 0.05 vs 0 h.

Schwann cells are an important part of the myelin sheath (22). In order to study the damage of EV71 to the myelin sheath, we infected Schwann cells with EV71 *in vitro* to observe the changes of Schwann cells. The MTS test showed that EV71 infection significantly inhibited cell proliferation, but this inhibitory effect weakened with time (Fig. 4A). EV71 infection significantly reduced ATP production at the time point of 48, 60, and 72 h. But at 84 h of infection, there were no differences in ATP production between the EV71 infected cells and the control group (Fig. 4B). Also, EV71 infection promoted cell apoptosis in a time-dependent manner, with the highest apoptosis level at 36 h of infection, followed by a gradual decrease in the level of apoptosis with the extension of time (Fig. 4C and D). After 60 h of infection, the apoptosis level did not significantly differ from that of the uninfected group. At the same time, these findings were confirmed by the TUNEL assay. At 36 h of EV71 infection, the level of cell apoptosis reached its peak and gradually decreased with prolonged time (Fig. 5A and B). This result indicates that the cell growth status gradually improved with the decrease of EV71 viral load.

### EV71 infection downregulates miR-29b expression and promotes PMP22 expression

**Fig 6:**
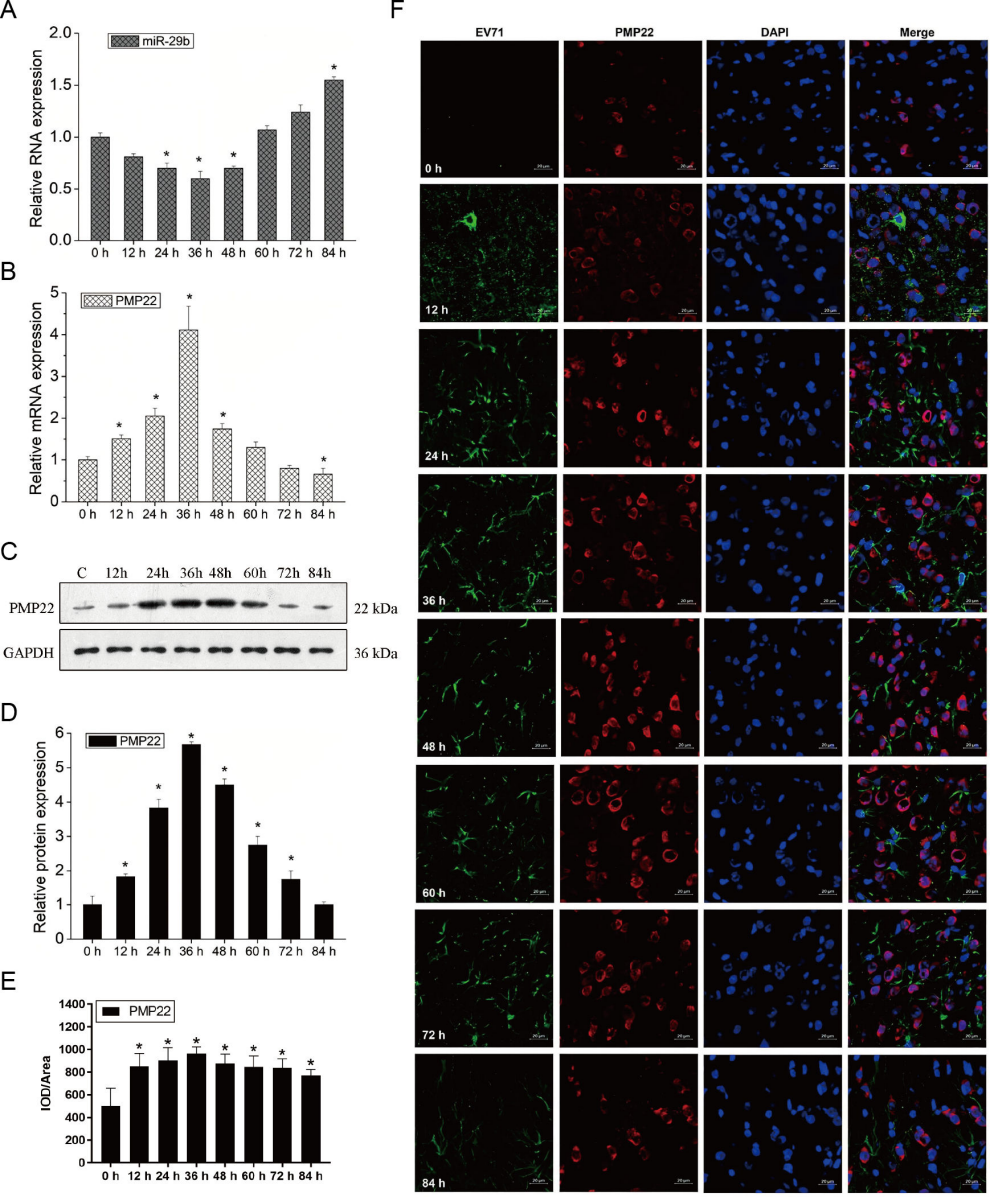
EV71 regulates the expression of miR-29b and PMP22. Schwann cells were collected after EV71 infection at a series of times from 0 to 84 h. (**A**) mRNA expression level of miR-29b at different time point. (**B**) mRNA expression level of PMP22 at different time point. (**C**) Representative image of PMP22 expression by western blot analysis. (**D**) Quantification of PMP22 expression level by western blot analysis. (**E**) Quantification of relative PMP22 expression level by immunofluorescence staining analysis. (**F**) Representative images of EV71 and PMP22 stain by immunofluorescence assay. Laser scanning confocal microscopy of Schwann cells stained with nuclear stain (DAPI, fluorescing blue), EV71 stain (fluorescing green), and PMP22 stain (fluorescing red). Scale bars are 20 µm. *P < 0.05 vs 0 h.

Some studies have found that the role of EV71 is related to PMP22, and miR-29a can target and regulate the expression of PMP22 (15, 16). However, sequencing analysis showed that miR-29b had a higher matching degree with PMP22 in miR-29 family. Therefore, this study further examined the relationship between EV71 and miR-29b/PMP22. EV71 inhibited the expression of miR-29b and promoted the expression of PMP22 in a time-dependent manner. At 36 h of EV71 infection, the expression level of miR-29b was the lowest; on the contrary, the expression level of PMP22 was the highest. Furthermore, After 36 h of EV71 infection, the expression of miR-29b was gradually increased, while the expression of PMP22 was gradually decreased (Fig. 6A and B). Western blot analysis also showed a similar result (Fig. 6C and D). While from the immunofluorescence detection, the average fluorescence intensity of PMP22 did not have a significant change along with the prolonged infection time (Fig. 6E and F). Therefore, these data suggest that EV71 can regulate the expression of miR-29b/PMP22 in Schwann cells.

### miR-29b can salvage PMP22 elevation caused by EV71 infection

**Fig 7:**
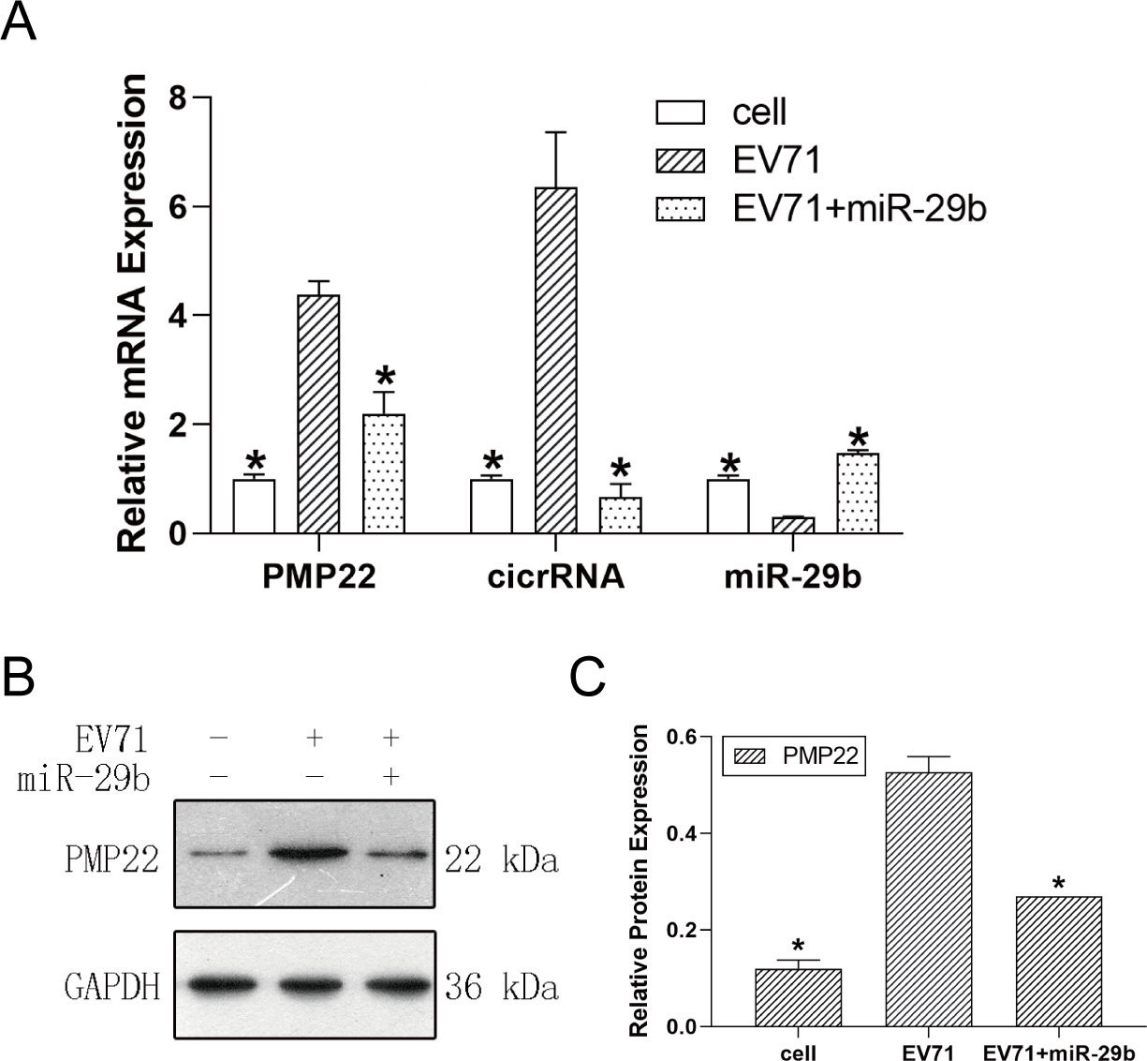
miR-29b can salvage PMP22 elevation caused by EV71 infection. Schwann cells were collected after EV71 infection at 36 h and divided into three groups: non-infected cell, EV71-infected cell, and EV71-infected cell with overexpression of miR-29b. (**A**) mRNA expression level of PMP22, cicrRNA, and miR-29b. (**B**) Representative image of PMP22 expression by western blot analysis. (**C**) Quantification of PMP22 expression level by western blot analysis. *P < 0.05 vs EV71-infected cell.

As we found that EV71 inhibited the expression of miR-29b and promoted the expression of PMP22 in previous studies, we further designed experiments to observe whether the increase of miR-29b expression could reduce the increase of PMP22 caused by EV71 infection. At the mRNA level, the expression of PMP22 and cicrRNA was significantly increased in EV71-infected cells. However, miR-29b overexpression reversed this phenomenon, resulting in the downregulated PMP22 and cicrRNA expression in EV71-infected cells (Fig. 7A). The expression of PMP22 at protein level also showed a similar result (Fig. 7B and C). Therefore, the high expression of miR-29b can reduce the high expression of PMP22 caused by EV71 infection.

### EV71 can affect the expression of hsa_circ_0069335

**Fig 8:**
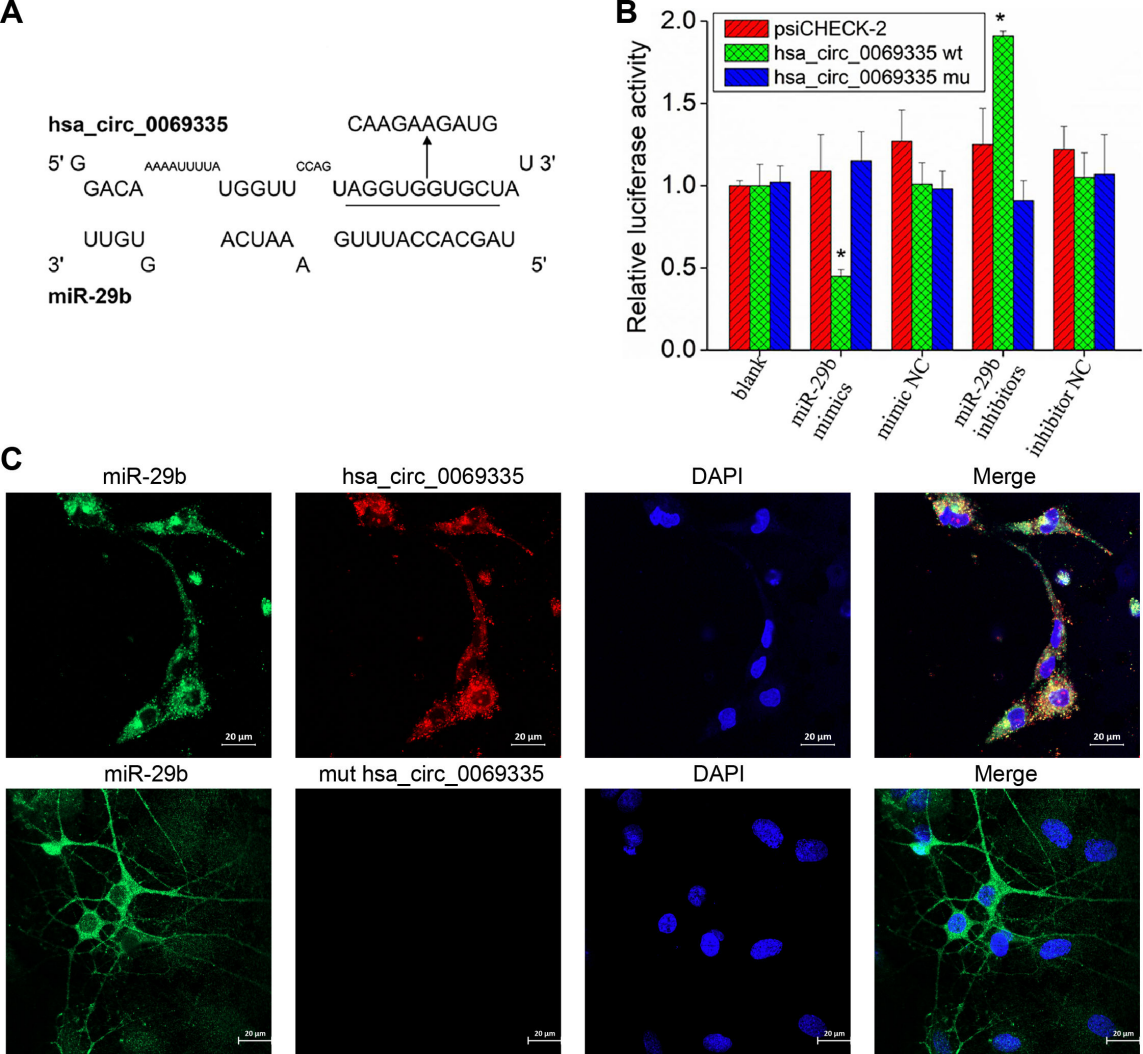
The regulatory relationship between hsa_circ_0069335 and miR-29b. (**A**) Potential-binding site of miR-29b in hsa_circ_0069335 sequence. The 5′-AGGUGGUGC-3′ sequence of binding site in hsa_circ_0069335 was changed to 5′-CAAGAAGAUG-3′ in the mutated hsa_circ_0069335. (**B**) The binding relationship between hsa_circ_0069335 and miR-29b detected by dual luciferase reporter gene assay. (**C**) Intracellular localization of hsa_circ_0069335 and miR-29b detected by FISH. Laser scanning confocal microscopy of Schwann cells stained with nuclear stain (DAPI, fluorescing blue), miR-29b stain (fluorescing green), and hsa_circ_0069335 stain (fluorescing red). Scale bars are 20 µm.* *P* < 0.05 vs psiCHECK-2.

In recent years, continuous studies have shown that circRNAs also have an important role in the progression of various diseases, which mainly exert through the adsorption of miRNAs (18, 19). Therefore, this study continued to explore the relationship between EV71 and circRNAs. The differentially expressed circRNAs in EV71 infection were identified by sequencing. Meanwhile, the circRNA, which could bind to miR-29b, was analyzed by software. The intersection of the two types of circRNAs was selected, among which hsa_circ_0069335 had the highest matching degree with miR-29b (Fig. 8A). A dual luciferase reporter gene experiment showed that miR-29b could bind to hsa_circ_0069335 and then significantly reduce luciferase activity (Fig. 8B). Fluorescence *in situ* hybridization (FISH) detection revealed that miR-29b and hsa_circ_0069335 were co-located and mainly located in the cytoplasm (Fig. 8C). Therefore, the function of hsa_circ_0069335 is related to miR-29b and could be regulated by EV71.

## DISCUSSION

So far, research on the pathogenic mechanism of EV71 and the screening of drugs and vaccines are still in the early stages for clinical application. Currently, there is no specific therapy for EV71 infection, and there is a lack of simple, feasible, and reliable biomarker for nerve injury diagnosis (23). The present study investigated the pathological changes in mice caused by EV71 infection, demonstrating that EV71 infection inhibits Schwann cell growth. Moreover, with the decrease of EV71 viral load, the cell growth status gradually improved. EV71 infection inhibits the expression of miR-29b and then promotes the expression of PMP22 in Schwann cells, and miR-29b can salvage PMP22 elevation caused by EV71 infection. Furthermore, we found that EV71 can affect the expression of hsa_circ_0069335, which is co-located with miR-29b. These results suggest that the miR-29b/PMP22 axis may serve as a novel potential therapeutic target against EV71-induced neuronal disorder in severe HFMD cases and provide evidence that circRNAs may be involved in the process.

This study found that EV71 infection resulted in vacuolar degeneration and contraction of neurons, looseness and edema of brain tissue, and decreased number of Nissl bodies. Schwann cells were swollen, dissolved, and showed apparent structural damage, and the organelles in the cytoplasm were reduced. The number and thickness of intercellular myelin sheaths were decreased significantly, the structure of myelin lamella was not clear, and the morphology of myelinated nerve fibers was irregular and loosely arranged. This indicates that EV71 infection impairs the process of myelination and increases the risk of virus retrograde transport and damage to neuronal cell bodies, leading to neurological damage. However, with the extension of infection time, EV71 viral load was decreased, and the pathological damage to the nervous system was gradually reduced. Some research showed that capsid protein VP1 can bind to scavenger receptor class B member 2 (SCARB2) on the host nerve cell membrane. As a result, EV71 can lead to nervous system complications through direct nerve invasion, involving both peripheral and central nervous systems, as well as myelin sheaths and neurons (24, 25). Through the immunohistochemistry and *in situ* hybridization detection of fatal EV71 cases, Wong et al. (26) found that EV71 was mainly located in neurons and surrounding inflammatory cells in areas with obvious inflammation, such as brain stem and anterior horn of the spinal cord, and no evident EV71 expression was found in non-neuronal cells. So they speculate that the pathogenic pathway of EV71 may be the retro-infection of the central nervous system through the peripheral nerve. Through animal experiments, Chen et al. (27) also confirmed that retrograde axonal transport may be the main way for EV71 to affect the central nervous system. Few articles have reported the relationship between EV71 viral load and pathological change degree. A previous study found that different viral loads lead to different pathological changes in the brain, while high viral loads lead to more severe pathological damage than low viral loads (28). Our results also showed that the EV71 viral load can influence the degree of pathological changes in the nervous system when EV71 infects the mice for the first time, which provides a new perspective for decreasing the pathological damage in the nervous system caused by EV71 infection through lowering the viral load.

Infection of Schwann cells with EV71 showed that cell proliferation and ATP production were significantly reduced, while apoptosis was significantly increased. However, with the extension of infection time, the cell growth state was gradually improved. Another study found that EV71 induced the expression of Atg5, Atg7, and LC3 II to enhance Schwann cell autophagy and meanwhile promoted Bax expression and down-regulated Bcl-2 expression to induce cell apoptosis (29). Moreover, the knockdown of METTL3 decreased the viral titer of EV71 and reduced EV71-induced cell death. During the development of the peripheral nervous system, Schwann cells are the main myelin-forming cells, which wrap around axons to form myelin and secrete nutrient factors. They also have a role in supporting and maintaining the integrity of axons and rapidly transmitting nerve signals (30, 31). After peripheral myelin injury, Schwann cells proliferate and migrate to the injury site and then differentiate to form new myelin sheaths. EV71 infection can induce Schwann cell apoptosis and excessive autophagy, thereby inhibiting cell proliferation and hindering myelin sheath regeneration. In addition, EV71 inhibited the expression of miR-29b and promoted the expression of PMP22 in a time-dependent manner, with the most significant change at 36 h of infection. Subsequently, the expression of miR-29b and PMP22 was gradually restored with the decrease of EV71 viral load. This result indicates that the regulation of EV71 on the biological function of Schwann cells is related to miR-29b and PMP22. Stavrou et al. (32, 33) pointed out that overexpression of PMP22 protein activates the unfolded protein response, thereby promoting apoptosis of Schwann cells, leading to dysmyelination and demyelination, secondary axonal loss, and neurological dysfunction. Also, the increase of miR-29b expression could reduce the increase of PMP22 caused by EV71 infection, which indicated that EV71 may affect Schwann cell function by regulating mir-29b/PMP22 axis. Further research is still needed to validate the mechanism through which EV71 affects Schwann cell.

In addition, EV71 regulated the expression of hsa_circ_0069335, and hsa_circ_0069335 could bind and co-locate with miR-29b. From that, we speculated that EV71 can regulate the expression of PMP22 through hsa_circ_0069335/miR-29b, which in turn affects the biological function of Schwann cells. However, this conclusion needs to be experimentally confirmed. In another research (21), circ-Ankib1 circRNA could regulate the expression of a series of target genes by sponging miR-485–5p, miR-423–5p, and miR-666–3p, thus inhibiting Schwann cell proliferation and axon regeneration after sciatic nerve injury, which indicates that circRNAs can serve as potential therapeutic targets for nerve injury repair. Circ_0002538 upregulated the expression of plasmolipin by sponging miR-138–5p, thus promoting the migration of Schwann cells, alleviating demyelination, and improving sciatic nerve function (34). The hypoxic bone mesenchymal stem cell-derived exosomes were able to direct Schwann cells proliferation, migration, and paracrine to accelerate facial nerve regeneration via circRNA_Nkd2/miR-214–3p/MED19 Axis (35). Those studies all showed that some specific circRNAs have vital regulatory roles in Schwann cells. To the best of our knowledge, this is the first time that miR-29b/PMP22 and hsa_circ_0069335 were linked together, providing a new insight into hsa_circ_0069335/miR-29b/PMP22 pathway and new drug target for EV71 infection.

In conclusion, EV71 infection inhibited Schwann cell growth and impaired myelination, leading to significant damage of the nervous system, which may be related to hsa_circ_0069335/miR-29b/PMP22 pathway. This discovery may provide critical molecular targets for the early warning and treatment of severe HFMD, thereby accelerating the progress of drug development for the treatment of severe HFMD patients.
